# Meta-analysis approach as a gene selection method in class prediction: does it improve model performance? A case study in acute myeloid leukemia

**DOI:** 10.1186/s12859-017-1619-7

**Published:** 2017-04-11

**Authors:** Putri W. Novianti, Victor L. Jong, Kit C. B. Roes, Marinus J. C. Eijkemans

**Affiliations:** 1 Biostatistics & Research Support, Julius Center for Health Sciences and Primary Care, University Medical Center Utrecht, 3508 GA Utrecht, The Netherlands; 2 Department of Epidemiology and Biostatistics, VU University medical center, Amsterdam, The Netherlands; 3 Department of Pathology, VU University medical center, Amsterdam, The Netherlands; 4 Viroscience Laboratory, Erasmus Medical Center Rotterdam, 3015 CE Rotterdam, The Netherlands

**Keywords:** Meta-analysis, Gene expression, Predictive modeling, Acute myeloid leukemia

## Abstract

**Background:**

Aggregating gene expression data across experiments via meta-analysis is expected to increase the precision of the effect estimates and to increase the statistical power to detect a certain fold change. This study evaluates the potential benefit of using a meta-analysis approach as a gene selection method prior to predictive modeling in gene expression data.

**Results:**

Six raw datasets from different gene expression experiments in acute myeloid leukemia (AML) and 11 different classification methods were used to build classification models to classify samples as either AML or healthy control. First, the classification models were trained on gene expression data from single experiments using conventional supervised variable selection and externally validated with the other five gene expression datasets (referred to as the individual-classification approach). Next, gene selection was performed through meta-analysis on four datasets, and predictive models were trained with the selected genes on the fifth dataset and validated on the sixth dataset. For some datasets, gene selection through meta-analysis helped classification models to achieve higher performance as compared to predictive modeling based on a single dataset; but for others, there was no major improvement. Synthetic datasets were generated from nine simulation scenarios. The effect of sample size, fold change and pairwise correlation between differentially expressed (DE) genes on the difference between MA- and individual-classification model was evaluated. The fold change and pairwise correlation significantly contributed to the difference in performance between the two methods. The gene selection via meta-analysis approach was more effective when it was conducted using a set of data with low fold change and high pairwise correlation on the DE genes.

**Conclusion:**

Gene selection through meta-analysis on previously published studies potentially improves the performance of a predictive model on a given gene expression data.

**Electronic supplementary material:**

The online version of this article (doi:10.1186/s12859-017-1619-7) contains supplementary material, which is available to authorized users.

## Background

The ability of microarray technology to simultaneously measure expression values of thousands of genes has brought major advances. The measurement of gene expression may be done within a relatively short time to quantify genome-wide expression levels. On the other hand, statistical analyses to extract useful information from such high dimensional data face well known challenges. Common mistakes in conducting statistical analyses were reported [1]. Particularly class prediction studies are subject to concerns about reliability of results [2], where genes involved in predictive models depend heavily on the subset of samples used to train the models. This is related to the likelihood of false positive findings due to the curse of dimensionality in microarray gene expressions datasets [3].

Methods for aggregating gene expression data across experiments exist [4, 5]. Data standardization is proposed as a preliminary step in cross-platform gene expression data analyses [6–8], as raw gene expression datasets are recommended to be used [9] and gene expression values may be incomparable across different experiments. Meta-analysis is known to increase the precision of the effect estimate and to increase the statistical power to detect a certain effect size (or fold change). In class prediction, meta-analysis methods can have different objectives, ranging from methods for combining effect sizes [10] or combining P values [11, 12] to rank-based methods [13]. However, there is no meta-analysis method known to be generally superior to others [14, 15].

In this study, we compared the performance of classification models on a given gene expression dataset between gene selection through meta-analysis on other studies and conventional supervised gene selection. A single gene expression dataset with less than a hundred samples is likely not enough to determine whether a particular gene is an informative gene [16]. Thus, gene selection based on multiple microarray studies may yield a more generalizable gene list for predictive modeling. We used raw gene expression datasets from six published studies in acute myeloid leukemia (AML) to develop predictive models using 11 different classification functions to classify patients with AML versus normal healthy controls. In addition, a simulation study was conducted to more generally assess the added value of meta-analysis for predictive modeling in gene expression data.

## Methods

As a starting point, we assume *D* gene expression datasets are available for analysis. First, the *D* raw datasets are individually preprocessed. Next, 11 classifiers are trained on expression values from the *j* ^*th*^ study (*j* = 1, …, *D*) by incorporating variable selection procedure via limma method and externally validated on the remaining *D-1* gene expression datasets. We refer to these models as *individual-classification* models.

**Fig. 1:**
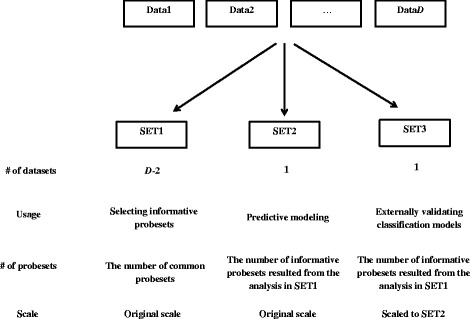
Data division to perform cross-platform classification models building and their characteristics. (#: the number)

To aggregate gene expression datasets across experiments, *D* gene expression datasets are divided into three major sets, namely (i) a set for selecting probesets (SET1, consists of *D-2* datasets), (ii) for predictive modeling using the selected probesets from SET1 (SET2, consists of one dataset) and (iii) for externally validating the resulting predictive models (SET3, consists of one dataset). The data division is visualized in Fig. 1. We next describe the predictive modeling with gene selection via meta-analysis (refer to as MA(meta-analysis)-classification model). First, significant genes from a meta-analysis on SET1 are selected. Next, classification models are constructed on SET2 using the selected genes from SET1. The models are then externally validated using the independent data in SET3. The MA-classification approach is briefly described in Table 1 and is elaborated in the next subsections.

**Table 1:** An approach in building and validating classification models by using meta-analysis as gene selection technique

1. Data collection
Collect raw gene expression datasets, which possibly come from previous experiments and/or systematic search from online repositories.
2. Data preparation
(i) Individually preprocess raw gene expression datasets (i.e. normalization, background correction, log2 transformation).
(ii) Divide *D* available gene expression datasets into three sets, i.e. *D-2* gene expression datasets to get a gene signature list (SET1), a gene expression set to train classification models (SET2) and a dataset to validate the models (SET3).
3. Meta-analysis for gene selection
(i) For each probesets, aggregate expression values from SET1 to get a signature list via random effect meta-analysis.
(ii) Record significant probesets (also refer to as informative probesets)
4. Predictive modeling
(i) In SET2, include informative probesets resulted from Step 3.
(ii) Divide samples in SET2 to a learning set and a testing set.
(iii) Perform cross validation in classification model modeling.
(iv) Evaluate optimum predictive models in the testing set.
5. External validation
(i) In SET3, include probesets that are informative from Step 3.
(ii) Scale gene expression values in SET3 with SET2 as a reference.
(iii) Validate classification models from Step 4 to the scaled gene expressions data in SET3.

### Data extraction

Raw gene expression datasets from six different studies were used in this study, as previously described elsewhere [16, 17], i.e. E-GEOD-12662 [18] (Data1), E-GEOD-14924 [19] (Data2), E-GEOD-17054 [20] (Data3), E-MTAB-220 [21] (Data4), E-GEOD-33223 [22] (Data5) and E-GEOD-37307 [23] (Data6). Five studies were conducted on Affymetrix Human Genome U133 Plus 2 array and one study was performed on U133A (Additional file 1: Table S1). The raw datasets were pre-processed by quantile normalization, background correction according to manufacturer’s platform recommendation, log_2_ transformation and summarization of probes into probesets by median polish to deal with outlying probes. We limited analyses to 22,277 common probesets that appeared in all studies.

### Meta-analysis for gene selection

We aggregated *D-2* gene expression datasets to extract informative genes by performing a random effects meta-analysis. This means meta-analysis acts as a dimensionality reduction technique prior to predictive modeling. For each probeset, we pooled the expression values across datasets in SET1 to estimate its overall effect size. Let *Y*_*ij*_ and *θ*_*ij*_ denote the observed and the true study-specific effect size of probeset *i* in an experiment *j*, respectively. The random effects model of a probeset *i* is written as:

$$ {Y}_{i j}={\theta}_{i j}+{\varepsilon}_{i j},\kern0.75em \mathrm{where}\ {\theta}_{i j}={\theta}_i+{\delta}_{i j}\kern0.5em \mathrm{f}\mathrm{o}\mathrm{r}\kern0.75em  i=1, \dots,\ p\kern0.5em \mathrm{and}\kern0.5em  j=1,\dots,\ \left( D-2\right), $$

where *p* is the number of tested probesets, *θ*_*i*_ is the overall effect size of probeset *i*, *ε*_*ij*_ ~ *N*(0; *σ*_*ij*_^2^) with *σ*_*ij*_^2^ as the within-study variance and *δ*_*ij*_ ~ *N*(0; *τ*_*i*_^2^) with *τ*_*i*_^2^ as the between-study or random effects variance of probeset *i*. The study-specific effect size *θ*_*ij*_ is defined as the corrected standardized mean different (SMD) between two groups, estimated by:

1 $$ {\hat{\theta}}_{ij}=\left(\frac{{\overline{x}}_{ij0}-{\overline{x}}_{ij1}}{s_{ij}}\right)\left(1-\frac{3}{4\left({n}_{j0}+{n}_{j1}\right)-9}\right), $$

where ${\overline{x}}_{ij0}\left({\overline{x}}_{ij1}\right)$ is the mean of base-2 logarithmically transformed expression values of probeset *i* in Group 0 (Group 1). *s*_*ij*_ is originally defined as the square root of the pooled variance estimate of the within-group variances [24]. This estimation of *σ*_*ij*_, however, is rather unstable in a small sample size study. We utilized the empirical Bayes approach implemented in limma to shrink extreme variances towards the overall mean variance. Thus, we define *s*_*ij*_ as the square root of the variance estimate from the empirical Bayes t-statistics [25]. The second component in Eq.(1) is the Hedges’ g correction for SMD [26]. The estimation of between-study variance $\left({\hat{\tau}}_i^2\right)$ was performed by Paule-Mandel (PM) method [27] as suggested by [28, 29]

For each probeset, a z-statistic was calculated to test the null hypothesis that the overall effect size in the random effects meta-analysis model is equal to zero (or a probeset is not differentially expressed). To adjust for multiple testing, P-values based on z-statistics were corrected at a false discovery rate (FDR) of *α* = 5%, using the Benjamini-Hochberg (BH) procedure [30]. We considered probesets that had a significant overall effect size as informative probesets. For each informative probeset *i,* the estimated overall effect size ${\theta}_i\ \left({\hat{\theta}}_i\right)$ is:

2 $$ {\hat{\theta}}_i=\frac{{\displaystyle {\sum}_j}{w}_{i j}{\hat{\theta}}_{i j}}{{\displaystyle {\sum}_j}{w}_{i j}}, $$

Where $$ {w}_{i j}=1/\left({\hat{\tau}}_i^2 + {s}_{i j}^2\right) $$.

### Classification model building

The following classification methods were used to construct predictive models: linear discriminant analysis (LDA), diagonal linear discriminant analysis (DLDA) [31], shrunken centroid discriminant analysis (SCDA) [32], random forest (RF) [33], tree-based boosting (TBB) [34], L2-penalized logistic regression (RIDGE), L1-penalized logistic regression (LASSO) [35], elastic net [36], feed forward neural networks (NNET) [37], support vector machines (SVM) [38] and k-nearest neighbors (kNN) [39]. A detailed description of the classification methods, model building procedure as well as the tuning -parameter(s) was presented in our previous study [40]. The class prediction modeling process for both individual- and MA-classification models was done by splitting the dataset in SET2 into a learning set ℒ and a testing set $\mathcal{T}$. The learning set ℒ was further split by cross validation into an inner-learning set and inner-testing set, to optimize the parameters in each classification model. The optimal models were then internally validated on the out-of-bag testing set $\mathcal{T}.$ Henceforth, we referred to the testing set $\mathcal{T}$ as an internal-validation set ${\mathcal{V}}_0$.

For MA-classification models on SET2, we used all the probesets identified as differentially expressed by meta-analysis procedure in SET1, except for LDA, DLDA and NNET methods, which cannot handle a larger number of parameters than samples. For these methods, we incorporated top-X probesets to the predictive modeling, where X was less than or equal to the sample size minus 1. The top lists of probesets were determined by ranking all significant probesets on their absolute estimated pooled effect sizes (${\hat{\theta}}_i$) from Eq.(2). As the number of probesets to be included was itself a tuning parameter, we varied the number of included probesets from 5 to the minimum number of within group samples. For other classification functions, we used the same values of tuning parameter(s) as described in our previous study [40].

For the individual-classification approach*,* we optimized the classification models based on a single gene expression dataset (SET2). Here, we applied the limma procedure [41] to determine top-X relevant probesets, controlling the false discovery rate at 5% using the BH procedure [30]. The optimum top-X was selected among{50, 100, 150, 200} for classification methods other than LDA, DLDA and NNET. We used the same number of selected probesets for the three aforementioned classification methods as in the MA-classification approach. In each case, we evaluated the classification models by the proportion of correctly classified samples to the number of total samples, known as a classification model accuracy.

### Model validation

The optimal classification models obtained from the previous step were externally validated on SET3. The log_2_ expression values of the data in SET3 for the probesets used in the classification models were scaled to the log_2_ expression values of the data in SET2, so that the learning and the validation sets had comparable range. For each probeset *i*, we assumed the expression values were in the interval [*a*_*i*_, *b*_*i*_] in SET2 and [*c*_*i*_, *d*_*i*_] in SET3. A log_2_ expression value *x*_*is*_ of probeset *i* in sample *s* from SET3, was scaled to the scale of SET2 by the following transformation formula:

3 $$ f\left({x}_{i s}\right)={a}_i + \frac{\left({b}_i-{a}_i\right)\left({x}_{i s}-{c}_i\right)}{\left({d}_i-{c}_i\right)},\kern0.75em {d}_i\ne {c}_i. $$

Predictive models were then applied to the scaled log_2_ gene expression data in SET3.

For individual-classification, we rotated the single learning dataset and validated the models on the other *D-1* datasets. For MA-classification, we rotated the datasets used for selecting informative probesets (SET1) as well as learning (SET2) and validating (SET3) classification models. For each possible combination of *D-2* datasets, we repeated step 3–5 of our approach (Fig. 1). Due to a small number of samples in Data3, we omitted the predictive modeling process when it was selected as SET2. Hence, the possible gene expression datasets in SET2 were Data1, Data2, Data4, Data5 and Data6; and gene expression datasets in SET3 were Data1, Data2, Data3, Data4, Data5 and Data6, rendering thirty possible combinations to divide *D = 6* datasets to three distinct sets.

### Simulation study

We generated synthetic datasets by conducting simulations similar to that described by Jong *et al* [42]. We refer to the publication for more detail description of each and every parameter stated in this sub-section. Among parameters to simulate gene expression data (Table 2, in [42]), we applied these following parameters for all simulation scenarios, i.e. (i) the number of genes per data set (*p* = 1000); (ii) the pairwise correlations of noisy genes were set equal to zero (implying **Σ**_33_ in Fig. 1. reference [42] was equal to 0), (iii) the proportion of differentially expressed genes (*π* = 10%) and; (iv) the parameter of an exponential distribution to draw the variances of the genes (*λ* = 0.5). Further, the number of samples per dataset (*n*), the log_2_ fold changes of differentially expressed (DE) genes (Δ) and pairwise correlations of DE genes (*ρ*) were varied as follows: *n* = 50, 100, 150; Δ = 0.1, 0.5, 0.75; and *ρ* = 0.25, 0.5, 0.75, respectively. We define pairwise correlation of noisy (*DE*) genes as the correlation between any and every two pairs of noisy (*DE*) genes. Table 2 shows nine combinations from these parameters, which reflect the amount of information in each simulated gene expression dataset. In the first block (simulation #1 to #3) for instance, the dataset generated by parameters in simulation #1 contains less information than the dataset generated by parameters in simulation #2, which is caused by the low degree of log_2_ fold changes and high correlation of DE genes.

**Table 2:** Parameters to generate simulated gene expression datasets

Simulation ID	*n*	Δ	*ρ*	DEG_MA_^a^	DEG_IND_^b^
1	50	0.1	0.75	12	72
2	50	0.5	0.5	57	34
3	50	0.75	0.25	70	62
4	100	0.1	0.75	12	14
5	100	0.5	0.5	53	56
6	100	0.75	0.25	67	50
7	150	0.1	0.75	15	23
8	150	0.5	0.5	52	26
9	150	0.75	0.25	58	57

Symbols. *n*: the number of samples in each generated dataset; Δ: the log_2_ fold changes of differentially expressed (DE) genes. *ρ*: pairwise correlation of DE genes

^a^The number of genes that were stated as differentially expressed (DE) genes by MA approach from 50 cumulative studies. All the selected genes are true positives

^b^The number of true DE genes among the top-100 DE genes selected by limma procedure

For each scenario mentioned in Table 2, we simulated data that consisted of n*52 samples from the same population. The data was then randomly divided into 52 different sub-datasets of *n* samples each (proportional to the classes). Next, the sub-datasets were randomly chosen to be considered as (i) SET1: a set of fifty datasets for selecting probes via meta-analysis; (ii) SET2: a dataset for predictive modeling; (iii) SET3: a dataset for validation. In the MA-predictive modeling, we estimated classification model accuracies when the number of studies for variable selection were ranging from 5 to 50 studies.

### Random effects linear regression

We quantified the difference in performance between classification models that were optimized with and without incorporating information from other studies in the simulation study by a random effects linear regression model. The difference of model accuracy between MA- and individual-classification procedure for a classification model *C* based on a simulation scenario *S* is denoted as *d*_*CSM*_. Such differences were calculated when MA-classification procedure incorporated *M* studies (where *M* = 5:50 by 5) to select features. Having rescaled the *d*_*CSM*_ to be in the range of 0 and 1 by $\frac{1+{d}_{CSM}}{2}$, we then transformed *d*_*CSM*_ using the logit function to get unbounded and more approximately normally distributed outcome values. Given in each simulation setting we calculated *d*_*CSM*_ for different number of *M* studies for feature selection in MA approach, we used a fully crossed random effects model, where simulation setting *S* and the number of studies for MA-approach *M* acted as clustering factors or random effects. Additionally, since the same classification methods were applied to build prediction models, classifier *C* was added as a random effect term.

We then tested three determinants (*X*_*k*_, *k* = 1, 2, 3) that might contribute to the difference in performance of classification models that were trained by two approaches (*d*_*CSM*_), namely the number of samples per dataset (*n*), the log_2_ fold changes of differentially expressed (DE) genes (Δ) and pairwise correlations of DE genes (*ρ*). Each of the determinant was individually evaluated in the random effects model. More formally, the random effects model for the *k* ^*th*^ determinant is written as:

$$ d{\hbox{'}}_{CSM}={\beta}_0+{\vartheta}_{0 C}+{\vartheta}_{0 S} + {\vartheta}_{0 M(S)} + {\beta}_1{X}_k, $$

where *d* ' _*CSM*_ is the logit transformation of the scaled *d*_*CSM*_; *ϑ*_0*S*_, *ϑ*_0*M*(*S*)_ and *ϑ*_0*C*_ are the random intercepts with respect to the simulation setting *S* (*ϑ*_0*S*_ ~ *N*(0, *σ*_0*S*_^2^)), the number of studies for meta-analysis *M* ( *ϑ*_0*M*(*S*)_ ~ *N*(0, *σ*_0*M*_^2^)) and classification model *C* (*ϑ*_0*C*_ ~ *N*(0, *σ*_0*C*_^2^)) respectively*.*

### Software

All analyses were performed in R statistical software using these packages: *affy* for preprocessing procedures [43]; *meta* for meta-analysis [44], *CMA* for predictive modeling [45], *lme4* for the random effects linear model [46] and *ggplot2* for data visualization [47].

## Results

**Fig. 2:**
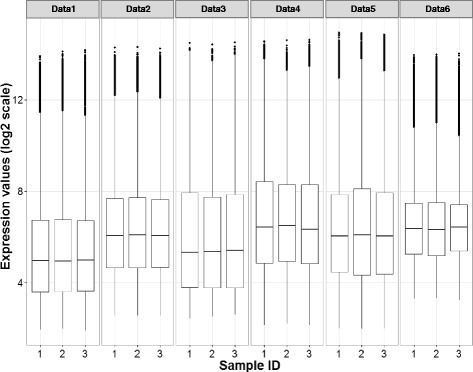
The distribution of expression values after pre-processing step from the first three samples in six experiments. The expression values are in log_2_ scale

We first present the performance of classification models when each individual study was used to optimize the classification functions (individual-classification procedure) in AML datasets. As the first illustration, we considered the case for which Data1 was used for optimization. To start with, we compared the distribution of expression values in the validation sets Data2 to Data6 to the expression values in Data1. There seemed to be a considerable difference in the distributions of expression values between studies, with Data6 having a lower range than other experiments, indicating that data standardization across studies was necessary (Fig. 2). Gene expression values in Data2 to Data6 were effectively scaled by using Eq.(3) so that they had comparable ranges as in Data1 (Additional file 1: Figure S1). The classification models optimized in Data1, were validated with Data2 to Data6. The classification models performed poorly in all 5 validation sets, notably worst in Data2 and Data4 (Additional file 1: Table S2). When Data2, Data4, Data5 and Data6 were used to optimize the classifiers, we found similar results (Additional file 1: Table S3-S6).

**Fig. 3:**
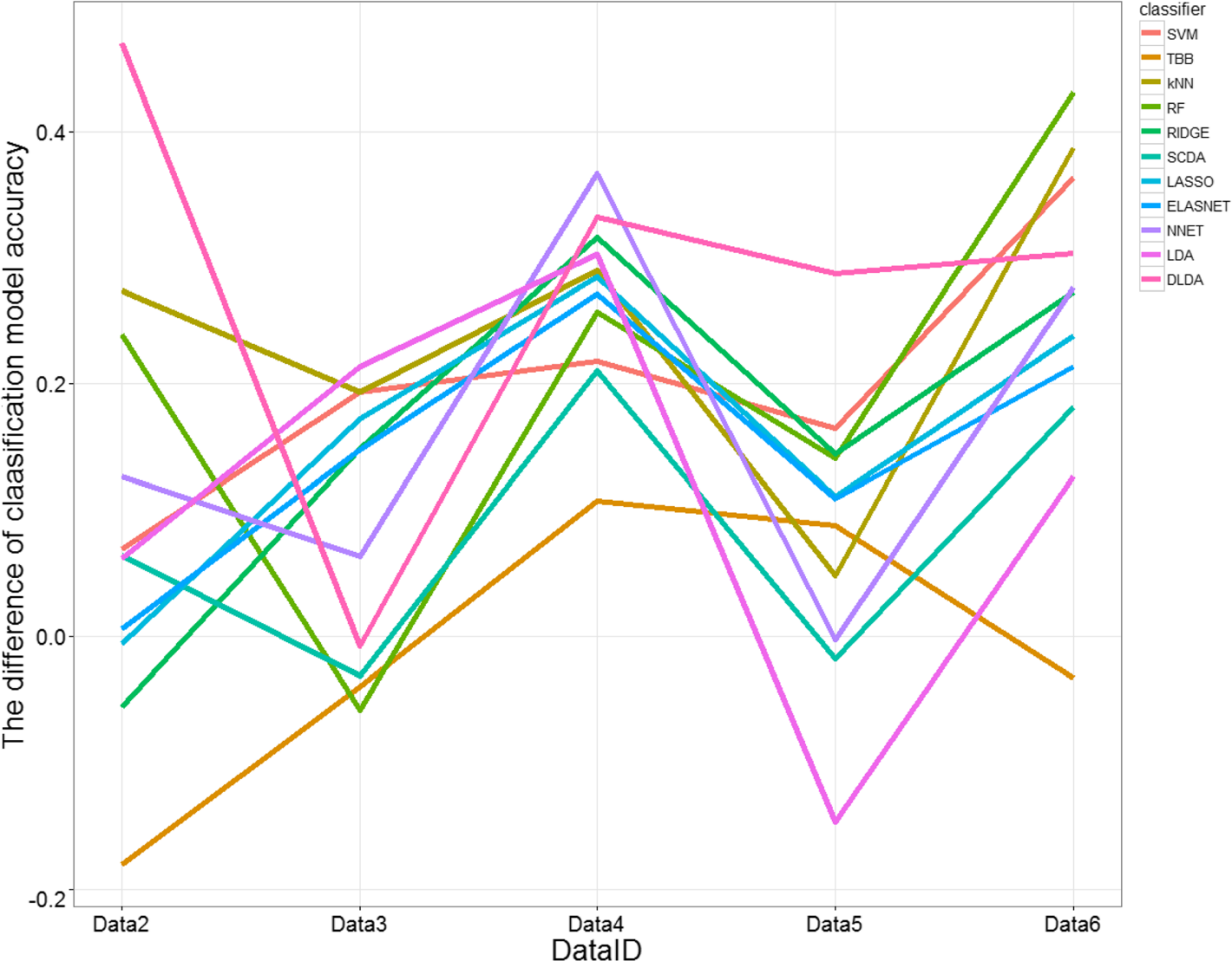
Plot of the difference of classification model accuracies between MA- and individual-classification approach, when Data1 was used as a training data

The comparison of the accuracies of classification models that were trained by MA- with individual-classification procedures based on optimization with Data1 is shown in Fig. 3. In most cases, MA-classification models outperformed individual-classification models. The difference of model accuracies between MA- and individual-classification approach was considerably larger when Data2 was used as a validation set. On average, classification methods that require the number of features to be smaller than the number of samples (i.e. NNET, LDA and DLDA), seemed to improve with the MA-classification approach. When validated against Data4, all models seemed to benefit from the MA-classification approach.

**Fig. 4:**
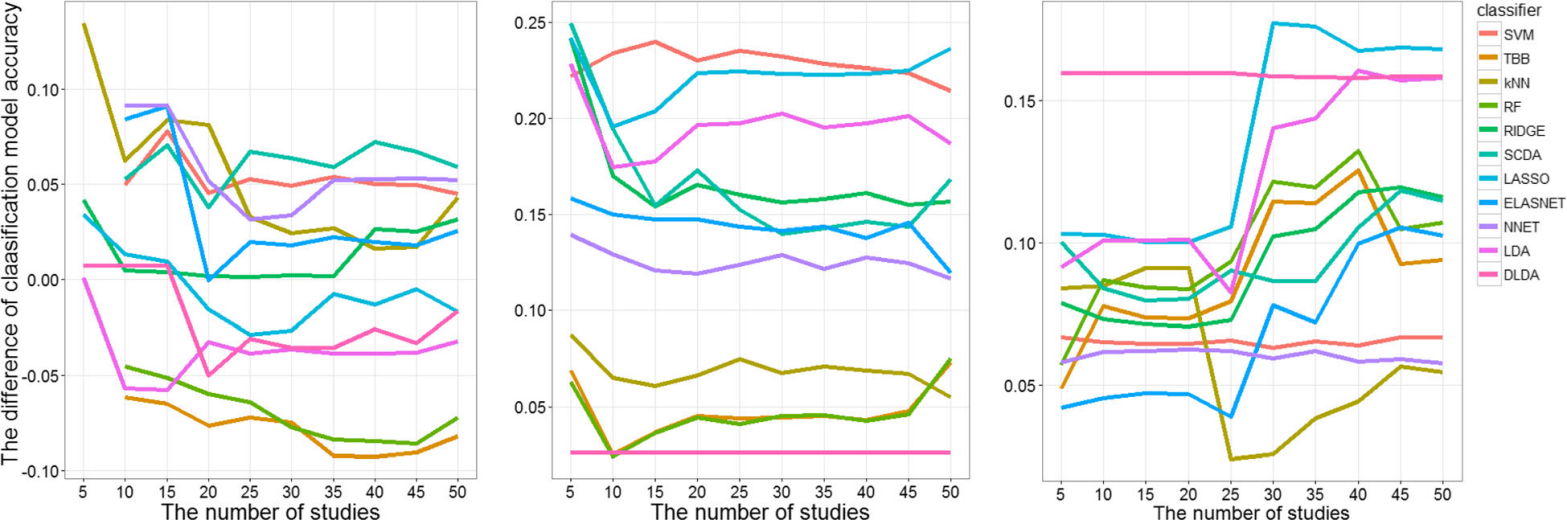
Plot of the difference of classification model accuracies between MA- and individual-classification approach in the simulated datasets, when Δ = 0.1, *γ* = 0.75 and (a) *n* = 50 (Simulation 1) (b) *n* = 100 (Simulation 4) (c) *n* = 150 (Simulation 7). The aforementioned simulation parameters resulted in the less informative datasets

In the other cases (i.e. when Data4, Data5 and Data6 acted as a learning set), we noticed that MA-classification approach did not outperform the individual-classification models when the models were validated on Data2. The MA-classification approach reduced the classification model accuracies by up to 50%, as compared to individual-classification models. As the MA-classification approach mostly resulted in a lower number of genes used in the predictive models than individual-classification approach, it might be hard for MA-classification models to outperform individual-classification models when validated on Data2, as DE genes in this dataset (on average) had a low degree of log_2_ fold change (i.e. 0.471). On the other hand, most of MA-classification models outperformed individual-classification models when they were validated on Data3 (Additional file 1: Figure S2-S5). Given that (i) the MA-approach was better in selecting the “true” DE genes (results from the simulation study) and more importantly (ii) the average log_2_ fold change of the DE genes in Data3 was considerably high, i.e. 2.025, in most cases the classifiers benefited from the MA-approach. Incorporating information from other experiments in these datasets did not consistently improve the predictive ability of classification models when externally-validated. The simulation study was conducted to evaluate the difference of classification model accuracies between the MA- and individual-classification approach more generally. The results showed that the MA-classification approach was more likely to improve the classification model accuracy when it was conducted in a set of less informative datasets (Fig. 4). We defined a less informative dataset as a dataset with a small number of samples, a low degree of log_2_ fold changes of the DE genes and a high level of pairwise correlation of DE genes. In this type of dataset, feature selection via limma method hardly selected the true DE genes in the individual-classification approach. Among the true 100 DE genes in each simulated dataset, the limma procedure could select 14 to 72 DE genes. Meanwhile, all selected genes by MA approach were truly DE genes (Table 2). As we observed in the AML data, classification methods that require the number of features less than the number of samples (i.e. NNET, LDA and DLDA) performed better with the feature selection prior to predictive modeling via meta-analysis.

Factors that might contribute to the difference of classification model accuracy between the MA- and individual-classification approach, were individually evaluated by random effect models. This resulted in the log_2_ fold changes and pairwise correlation between DE genes as the significant factors. Both factors were consistent with the finding that a set of less informative datasets benefited from the MA-classification approach (shown by negative coefficient on Δ and positive coefficient on *ρ*). Further, there was no additional variation in the difference in performance between MA- and individual-classification approach that was associated with the number of datasets used to select features in meta-analysis approach (*σ*_0*M*_^2^ = 0). A possible explanation of this finding could be that five datasets used in MA-classification approach were enough to select relevant variables so that the quality of the variable selection was not further increased by the increasing the number of datasets. This might also explain all the true positive genes selected by MA-approach in the simulation study. (Table 3)

**Table 3:** Results of the random effects models

Factors	Coefficient	Confidence interval		*σ*_0*C*_	Confidence interval		*σ*_0*S*_	Confidence interval		*σ*_0*M*(*S*)_	Confidence interval	
		LL	UL		LL	UL		LL	UL		LL	UL
*n*	0.0005	-0.0005	0.0009	0.0244	0.0165	0.0404	0.0489	0.0289	0.0759	0.000	0.000	0.0039
Δ	-0.1169	-0.2041	-0.0285	0.0245	0.0163	0.0402	0.0359	0.0159	0.0405	0.000	0.000	0.0039
*ρ*	0.1489	0.0295	0.2636	0.0245	0.0165	0.0405	0.0369	0.0022	0.0579	0.000	0.000	0.0039

Each factor was evaluated individually in the random effects linear regression model. The coefficients were inverse transformed to the original scale of the difference of classification model accuracy between MA- and individual classification approach

*Abbreviations*: *LL* lower limit, *UL* upper limit

Symbols: *n*: the number of samples in each generated dataset; Δ: the log2 fold change of differentially expressed (DE) genes. *ρ*: pairwise correlation of DE genes. *σ*_0*C*_, *σ*_0*S*_ and *σ*_0*M*(*S*)_ are the standard deviation of the random intercepts with respect to classification model, scenario in the simulation study and the number of studies used for selecting relevant features via meta-analysis approach. See Method section for more details regarding the random effect models

## Discussion

This study applied a meta-analysis approach for feature selection in predictive modeling on gene expression data. Selecting informative genes among massive noisy genes in predictive modeling faces a great challenge in microarray gene expression data. Dimensionality reduction is applied to reduce the number of noisy genes as well as to reduce the possibility of predictive models choosing clinically irrelevant biomarkers. An extra step to generate a gene signature list is usually applied in practice (e.g. by [48–53]), including predictive modeling via embedded classification methods (e.g. SCDA and LASSO). Selected informative genes may depend on the sub-samples used in the analysis [2], which may lead to the lack of direct clinical application [54].

Previous research on the application of meta-analysis in differential gene expression analysis showed that a single study might not contain enough samples to make a conclusion whether a particular gene is an informative gene. Among 12,211 common genes from 271 combined samples, 70 to 90% of the genes needed more samples in order to draw a conclusion [16]. A very low sample size as compared to the number of genes can cause false positive finding [3]. Involving thousands of samples is a straight forward solution but it can be very costly and time consuming. A possible solution to increase the sample size is by combining gene expression datasets with a similar research question through meta-analysis.

Meta-analysis is known as an efficient tool to increase statistical power and to obtain more generalizable results. Although a number of meta-analysis methods have been used as a feature selection technique in class prediction, no method has been shown to perform better than others [14, 17]. In this study, we combined the corrected standardized effect size for each gene by random effects models, similar to a study conducted by Choi *et al* [10]. However, we estimated the between-study variance by Paule-Mandel method, which outperforms the DerSimonian-Laird method in continuous outcome data [28]. We used a broad selection of classification functions to build predictive models in order to evaluate the added value of meta-analysis in aggregating information from gene expression across studies.

Six raw gene expression datasets resulting from a systematic search in a previous study in acute myeloid leukemia (AML) [16] were preprocessed, 22,277 common probesets were extracted and used for further analyses. We assessed the performance of classification models that were trained by each single gene expression dataset. The models were then validated on datasets obtained from other studies. Classification models that were externally validated might suffer from heterogeneity between datasets, due to, for instance, different sample characteristics and experimental set-up.

For some datasets, gene selection through meta-analysis yielded better predictive performance as compared to predictive modeling on a single dataset, but for others, there was no major improvement. Evaluating factors that might account for the difference in performance of the two predictive modeling approaches on real-life datasets could be confounded by uncontrolled variables in each dataset. As such, we empirically evaluated the effects of fold change, pairwise correlation between DE genes and sample size on the added value of meta-analysis as a gene selection method in class prediction with gene expression data.

The simulation study was performed to evaluate the effect of the level of information contained in a gene expression dataset. For a given number of samples, we defined an informative gene expression data as a dataset with large log_2_ fold changes and low pairwise correlation of DE genes. The simulation study shows that the less informative datasets (i.e. Simulation 1, 4 and 6) benefited from MA-classification approach more clearly, than the more informative datasets. The limma feature selection method on a single dataset had a higher false positive rate of DE genes compared to feature selection via meta-analysis. Incorporating redundant genes in the predictive model may weaken the performance of a classification model on independent datasets. While conventional procedures use the same experimental data, meta-analysis uses a number of datasets to select features. Thus, the chances of sub-samples-dependent features to be included in a predictive model are reduced in MA- than in individual-classification approachand the gene signature may be widely applied.

For MA, we defined the effect size as a standardized mean difference between two groups. Although we individually selected differentially expressed probesets (i.e. ignoring correlation among probesets), we incorporated information from all probesets by applying limma procedure in estimating the within-group variances (Eq.(1)). This empirical Bayes moderated t-statistics produces stable variances and it is proven to outperform ordinary t-statistics [55]. Marot *et al* implemented a similar approach in estimating unbiased effect sizes (Eq.(13) in [56]) and they suggested to apply such approach to estimate the study-specific effect size in meta-analysis of gene expression data.

We analyzed gene expression data at the probeset level. When more heterogeneous gene expression data from different platforms are used, mapping probesets to the gene level is a good alternative. Annotation packages from Bioconductor [57] and methods to deal with multiple probesets referring to the same gene may be considered, if such mapping is applied in a cross-platform gene expression study. A point to consider in cross-platform analysis of microarray experiments is data standardization. The same genes may have different signal in different experiments, due to e.g. different array technology and scanning process. We investigated the distributions of expression values across experiments and found incomparable ranges of expression values across experiments. Despite its simple nature, the scaling formula in Eq.(3) produces common ranges of gene expression values across experiments. Some methods to scale gene expression across experiments were proposed [7, 8, 10]. We do not expect that different scaling methods give significantly different findings as presented here, although it may be interesting to study.

We individually pre-processed the selected gene expression datasets, adjusted by the microarray platform in each and every study. A different preprocessing method may lead to different results of the prediction models, but it is not covered in this study. The predictive ability of a classification model may depend on a set of samples that is used in the preprocessing and normalization step. The rank-based genes is preferred over raw expression values to generate gene expression data [57]. Although we do not expect the present conclusions to change, it could be interesting to investigate this procedure further in this context.

## Conclusions

A meta-analysis (MA) approach was applied to select relevant features from multiple studies. Based on the simulation study, the MA approach was better in terms of variable selection than the predictive modeling by using a single dataset. In particular, a less informative dataset (which contains low log_2_ fold changes and highly correlated differentially expressed genes) was likely to benefit from feature selection via meta-analysis for class prediction. This also held for classification methods that require a smaller number of features than samples. Given the present public availability of omics datasets, meta-analysis approach can be used more often as an alternative gene selection method in class prediction.

## Additional files

Additional file 1: A supplementary material file. (PDF 747 kb)

Additional file 2: R scripts. (ZIP 11 kb)

Additional file 3: The simulated datasets. This folder contains synthetic datasets that were generated by using parameters described in Table 1. (ZIP 341408 kb)

## Abbreviations

AML: Acute myeloid leukemia
DE: Differentially expressed
DLDA: Diagonal linear discriminant analysis
kNN: k-nearest neighbors
LASSO: L1-penalized logistic regression
LDA: Linear discriminant analysis
MA: Meta-analysis
NNET: Feed forward neural networks
RF: Random forest
RIDGE: L2-penalized logistic regression
SCDA: Shrunken centroid discriminant analysis
SMD: Standardized mean difference
SVM: Support vector machines
TBB: Tree-based boosting
