# Supplementary material for: Meta-analysis approach as a gene selection method in class prediction: does it improve model performance? A case study in acute myeloid leukemia
Source: BMC Bioinformatics. 2017 Apr 11;18:210. doi: 10.1186/s12859-017-1619-7 (PMC5387259; doi:10.1186/s12859-017-1619-7)
Supplement: Supplementary file 1 — A supplementary material file. (PDF 747 kb) [file 12859_2017_1619_MOESM1_ESM.pdf]

Supplementary materials for

Meta-analysis approach as a gene selection method in class prediction: Does it improve model performance?

A case study in acute myeloid leukemia

Putri W Novianti[1,2,3], Victor L Jong [1,4], Kit CB Roes [1], Marinus JC Eijkemans[1]

[1] Biostatistics & Research Support, University Medical Center Utrecht, the Netherlands
[2] Epidemiology & Biostatistics department, VU University Medical Center Amsterdam, the Netherlands
[3] Pathology department, VU University Medical Center Amsterdam, the Netherlands
[4] Viroscience Lab, Erasmus Medical Center Rotterdam, the Netherlands

CONTENT

Supplementary tables

Table S1. Characteristics of the selected microarray gene expression studies

Table S2. The classification model accuracies of individual classification models that were optimized on Data1 and validated on the other datasets

Table S3. The classification model accuracies of individual classification models that were optimized on Data2 and validated on the other datasets

Table S4. The classification model accuracies of individual classification models that were optimized on Data4 and validated on the other datasets

Table S5. The classification model accuracies of individual classification models that were optimized on Data5 and validated on the other datasets

Table S6. The classification model accuracies of individual classification models that were optimized on Data6 and validated on the other datasets

Supplementary figures

Figure S1. The distribution of scaled expression values (log_2 scale) from the first three samples in six experiments, when Data1 was used as the reference data

Figure S2. Plot of the difference of classification model accuracies between MA- and individual-classification approach, when Data2 was used as a training data.

Figure S3. Plot of the difference of classification model accuracies between MA- and individual-classification approach, when Data4 was used as a training data.

Figure S4. Plot of the difference of classification model accuracies between MA- and individual-classification approach, when Data5 was used as a training data.

Figure S5. Plot of the difference of classification model accuracies between MA- and individual-classification approach, when Data6 was used as a training data.

Figure S6. Plot of the difference of classification model accuracies between MA- and individual-classification approach in the simulated datasets, when $\Delta = 0.5$, $\rho = 0.75$ and (a) $n = 50$ (Simulation 2) (b) $n = 100$ (Simulation 5) (c) $n = 150$ (Simulation 8). The aforementioned simulation parameters resulted in the medium informative datasets.

Figure S7. Plot of the difference of classification model accuracies between MA- and individual-classification approach in the simulated datasets, when $\Delta = 0.75$, $\rho = 0.25$ and (a) $n = 50$ (Simulation 3) (b) $n = 100$ (Simulation 6) (c) $n = 150$ (Simulation 9). The aforementioned simulation parameters resulted in the highly informative datasets.

SUPPLEMENTARY TABLES

Table S1. Characteristics of the selected microarray gene expression studies

Data ID	ArrayExpress ID	Year	Affy Platform	Sample size (Control; AML)	The initial number of probesets
1	E-GEOD-12662	2008	HG-U133 Plus 2	106 (30;76)	54675
2	E-GEOD-14924	2009	HG-U133 Plus 2	20 (10;10)	54675
3	E-GEOD-17054	2009	HG-U133 Plus 2	13 (4;9)	54675
4	E-MTAB-220	2011	HG-U133 Plus 2	43 (10;33)	54675
5	E-GEOD-33223	2012	HG-U133 Plus 2	30 (10;20)	54675
6	E-GEOD-37307	2012	HG-U133A	47 (17;30)	22283

Table S2. The classification model accuracies of individual classification models that were optimized on Data1 and validated on the other datasets

	Data2	Data3	Data4	Data5	Data6
SVM	0.331	0.729	0.596	0.501	0.530
TBB	0.595	0.671	0.641	0.495	0.692
kNN	0.326	0.768	0.597	0.652	0.576
RF	0.341	0.802	0.454	0.495	0.514
RIDGE	0.499	0.697	0.614	0.555	0.663
SCDA	0.367	0.754	0.468	0.722	0.580
LASSO	0.379	0.660	0.647	0.621	0.604
ELASNET	0.388	0.671	0.667	0.609	0.631
NNET	0.398	0.782	0.557	0.747	0.533
LDA	0.503	0.565	0.583	0.847	0.595
DLDA	0.300	0.662	0.394	0.546	0.474

Abbreviations: SVM: support vector machine; TBB: tree-based boosting; kNN: k-nearest neighbor; RF: random forest; SCDA: Shrunken centroid discriminant analysis (as known as predictive analysis of microarray, PAM); ELANET: elastic net; NNET: feed-forward neural networks; LDA: linear discriminant analysis; DLDA: diagonal linear discriminant analysis.

Table S3. The classification model accuracies of individual classification models that were optimized on Data2 and validated on the other datasets

	Data1	Data3	Data4	Data5	Data6
SVM	0.264	0.462	0.419	0.267	0.617
TBB	0.519	0.539	0.512	0.600	0.766
kNN	0.434	0.615	0.395	0.433	0.553
RF	0.305	0.462	0.433	0.382	0.631
RIDGE	0.387	0.615	0.326	0.400	0.596
SCDA	0.293	0.462	0.419	0.333	0.617
LASSO	0.340	0.692	0.326	0.600	0.575
ELASNET	0.346	0.678	0.349	0.561	0.619
NNET	0.375	0.462	0.429	0.476	0.589
LDA	0.236	0.692	0.488	0.400	0.532
DLDA	0.255	0.692	0.488	0.500	0.638

(Abbreviations are as in the Table S2)

Table S4. The classification model accuracies of individual classification models that were optimized on Data4 and validated on the other datasets

	Data1	Data2	Data3	Data5	Data6
SVM	1,000	0,761	0,564	0,668	0,753
TBB	0,615	0,783	0,650	0,633	0,766
kNN	1,000	0,746	0,601	0,699	0,683
RF	1,000	0,761	0,682	0,667	0,744
RIDGE	0,965	0,768	0,579	0,687	0,731
SCDA	1,000	0,764	0,696	0,731	0,806
LASSO	0,835	0,769	0,637	0,728	0,684
ELASNET	0,823	0,771	0,647	0,729	0,678
NNET	0,729	0,754	0,708	0,663	0,694
LDA	0,923	0,783	0,764	0,721	0,627
DLDA	1,000	0,768	0,844	0,700	0,757

(Abbreviations are as in the Table S2)

Table S5. The classification model accuracies of individual classification models that were optimized on Data5 and validated on the other datasets

	Data1	Data2	Data3	Data4	Data6
SVM	0.628	0.791	0.100	0.615	0.542
TBB	0.605	0.925	0.400	0.769	0.426
kNN	0.580	0.906	0.300	0.615	0.467
RF	0.548	0.892	0.198	0.754	0.427
RIDGE	0.448	0.914	0.146	0.678	0.426
SCDA	0.535	0.896	0.200	0.615	0.468
LASSO	0.698	0.858	0.500	0.660	0.483
ELASNET	0.698	0.877	0.500	0.692	0.426
NNET	0.707	0.883	0.500	0.692	0.539
LDA	0.698	0.915	0.450	0.769	0.489
DLDA	0.581	0.915	0.250	0.769	0.468

(Abbreviations are as in the Table S2)

Table S6. The classification model accuracies of individual classification models that were optimized on Data6 and validated on the other datasets

	Data1	Data2	Data3	Data4	Data5
SVM	0.764	0.792	0.716	0.988	0.972
TBB	0.733	0.717	0.850	1.000	0.900
kNN	0.713	0.809	0.568	0.706	0.922
RF	0.743	0.780	0.820	0.886	0.929
RIDGE	0.747	0.825	0.583	0.872	0.914
SCDA	0.637	0.767	0.803	0.917	0.950
LASSO	0.704	0.811	0.649	0.895	0.938
ELASNET	0.703	0.813	0.657	0.894	0.942
NNET	0.787	0.797	0.661	0.783	0.784
LDA	0.718	0.759	0.711	0.809	0.767
DLDA	0.769	0.767	0.661	0.835	0.887

(Abbreviations are as in the Table S2)

SUPPLEMENTARY FIGURES

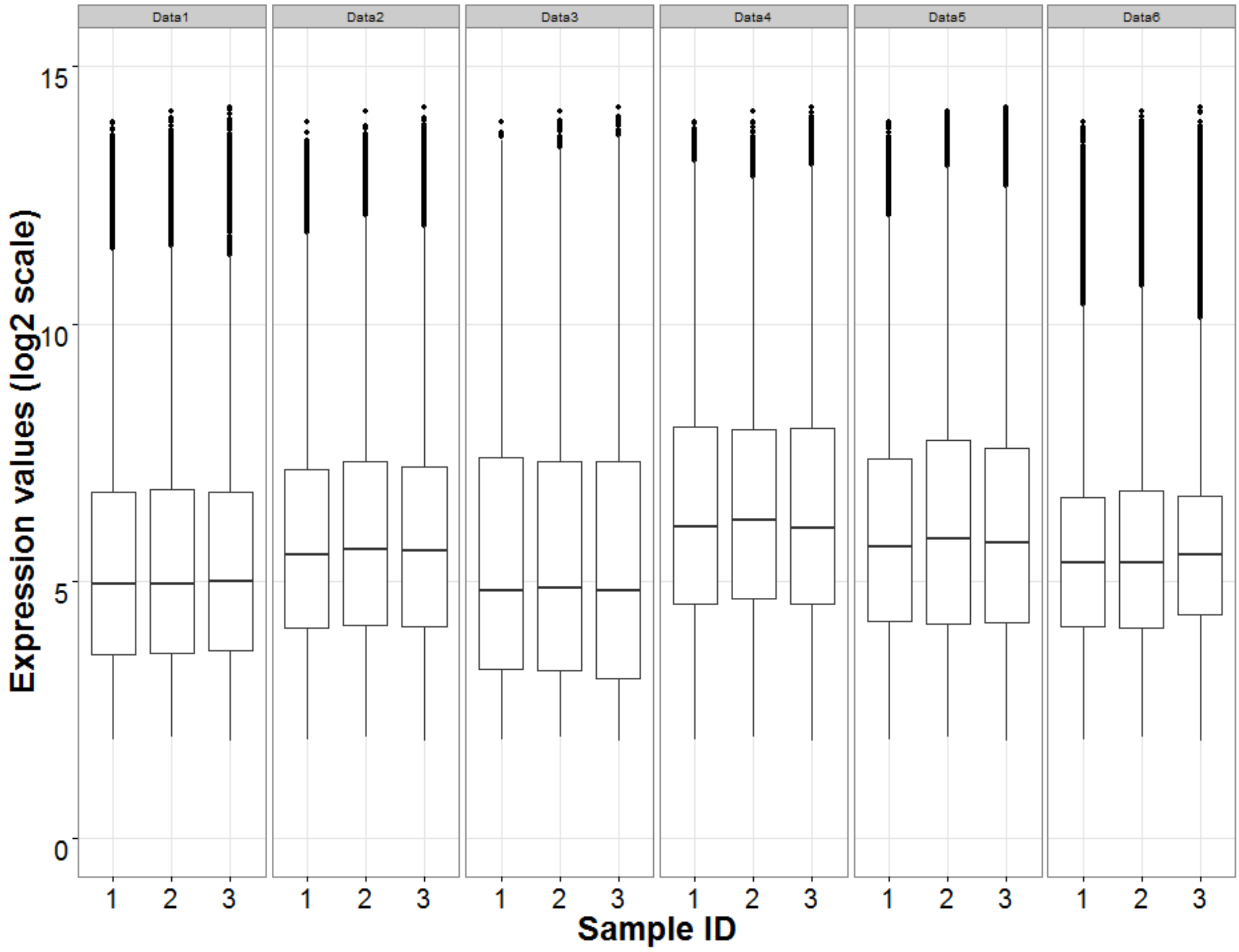

Figure S1. The distribution of scaled expression values (log_2 scale) from the first three samples in six experiments, when Data1 was used as the reference data

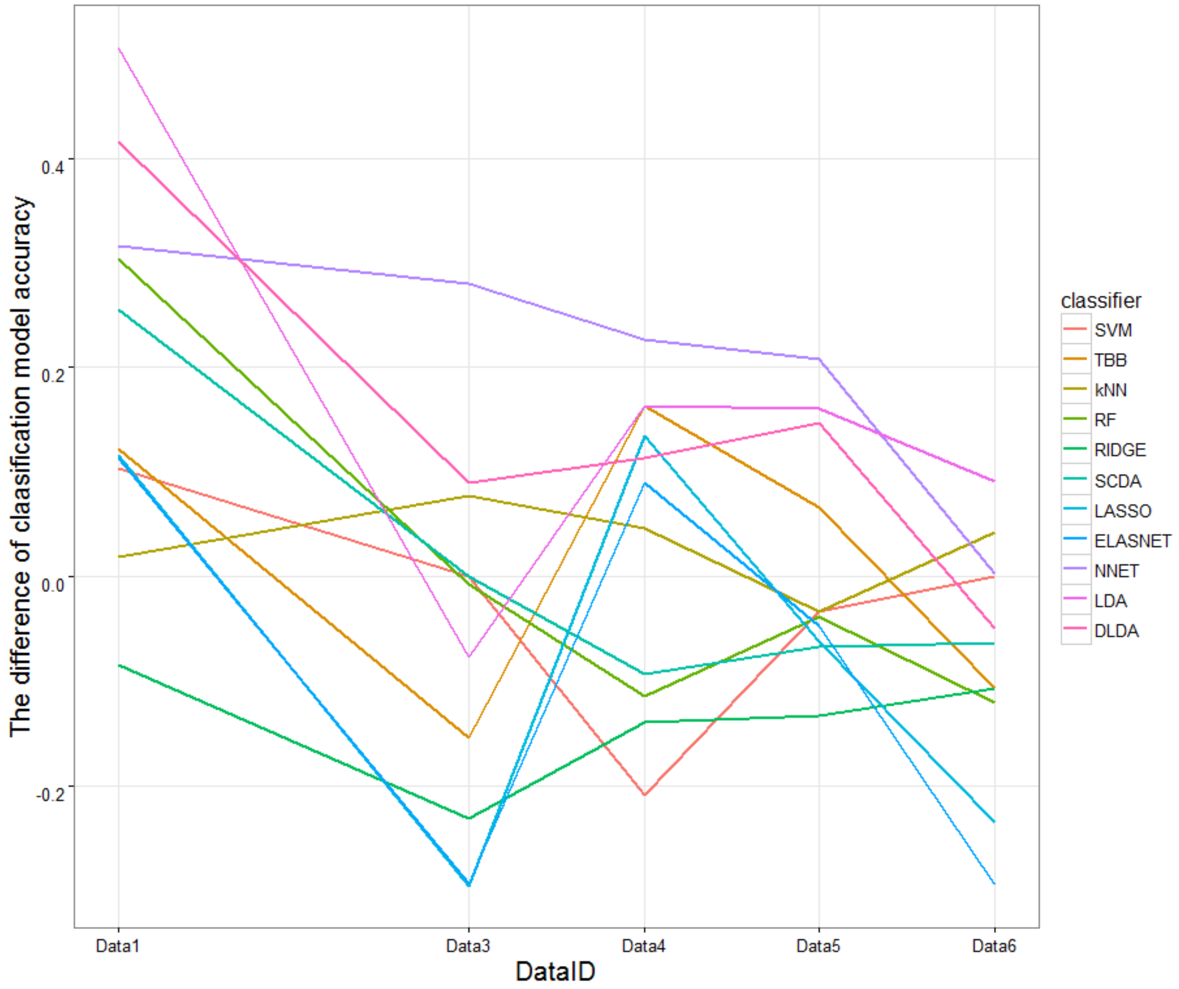

Figure S2. Plot of the difference of classification model accuracies between individual- and MA-classification approach, when Data2 was used as a training data.

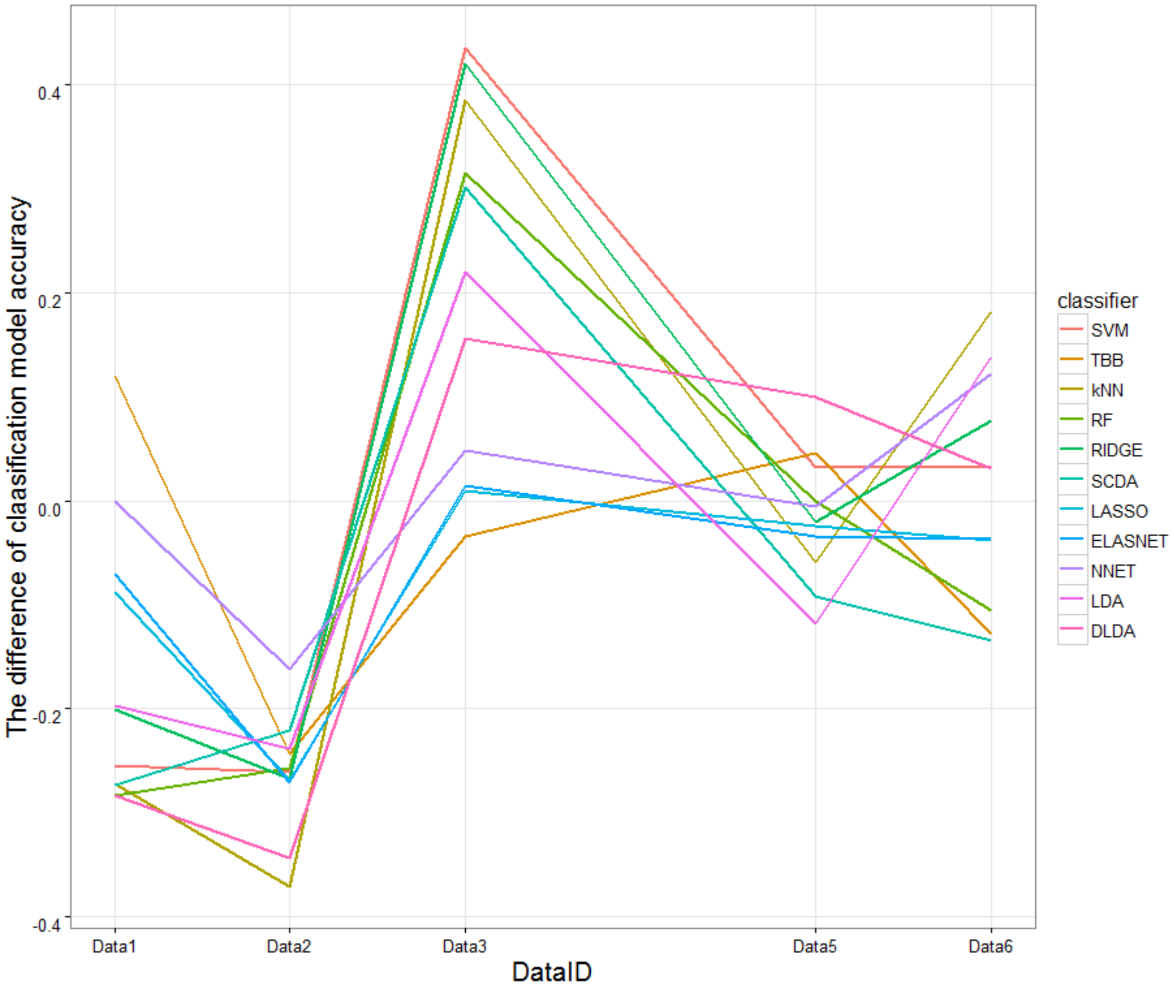

Figure S3. Plot of the difference of classification model accuracies between individual- and MA-classification approach, when Data4 was used as a training data.

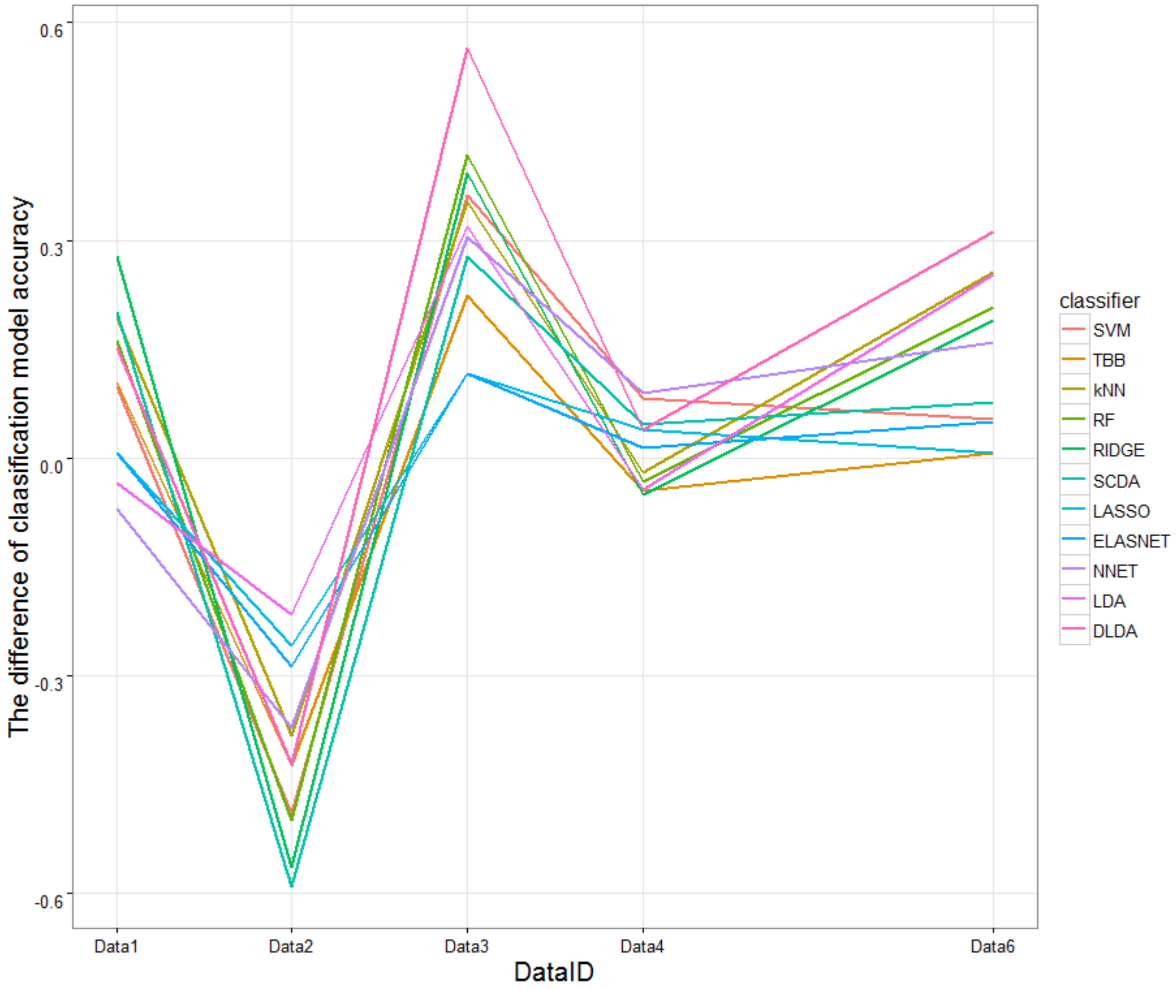

Figure S4. Plot of the difference of classification model accuracies between individual- and MA-classification approach, when Data5 was used as a training data.

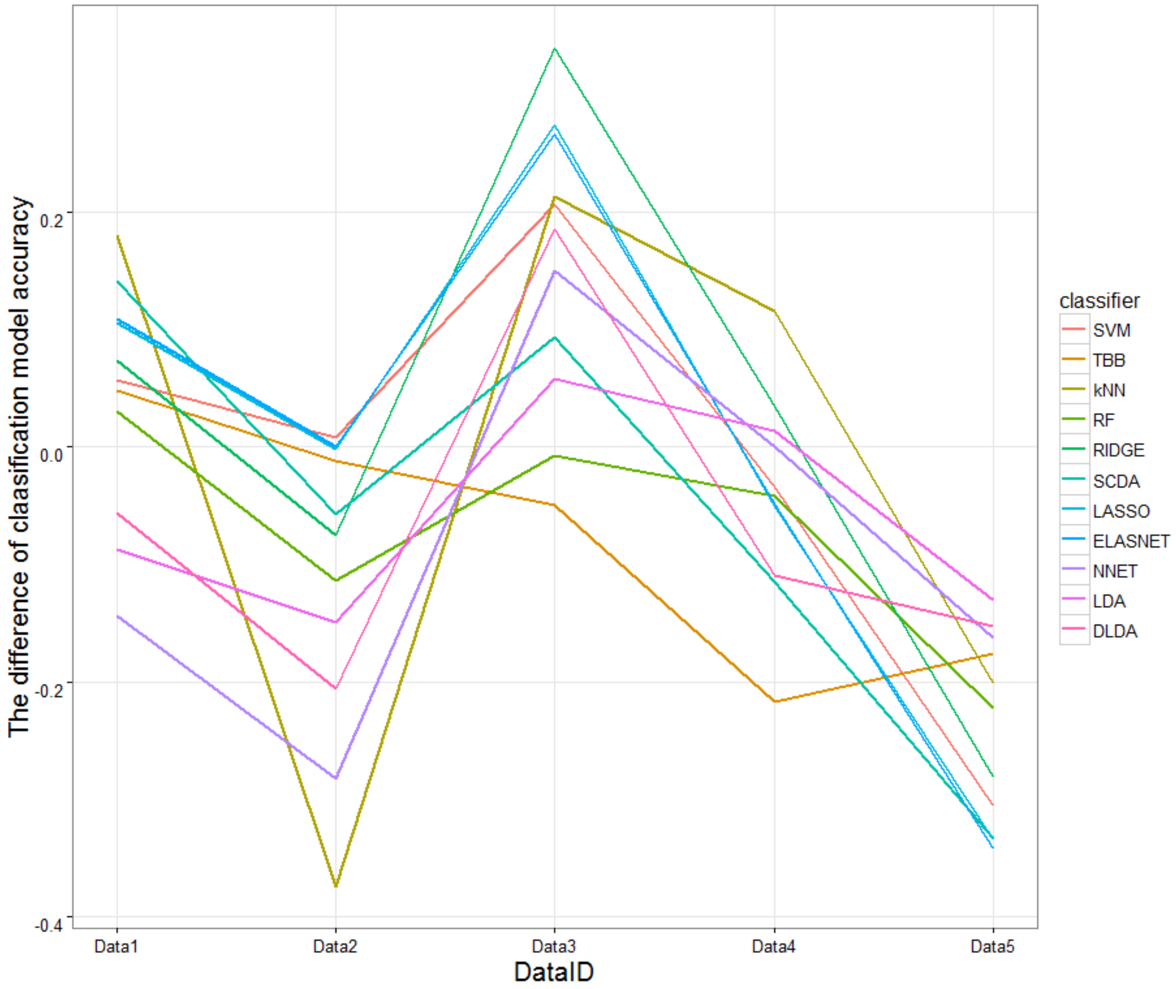

Figure S5. Plot of the difference of classification model accuracies between individual- and MA-classification approach, when Data6 was used as a training data.

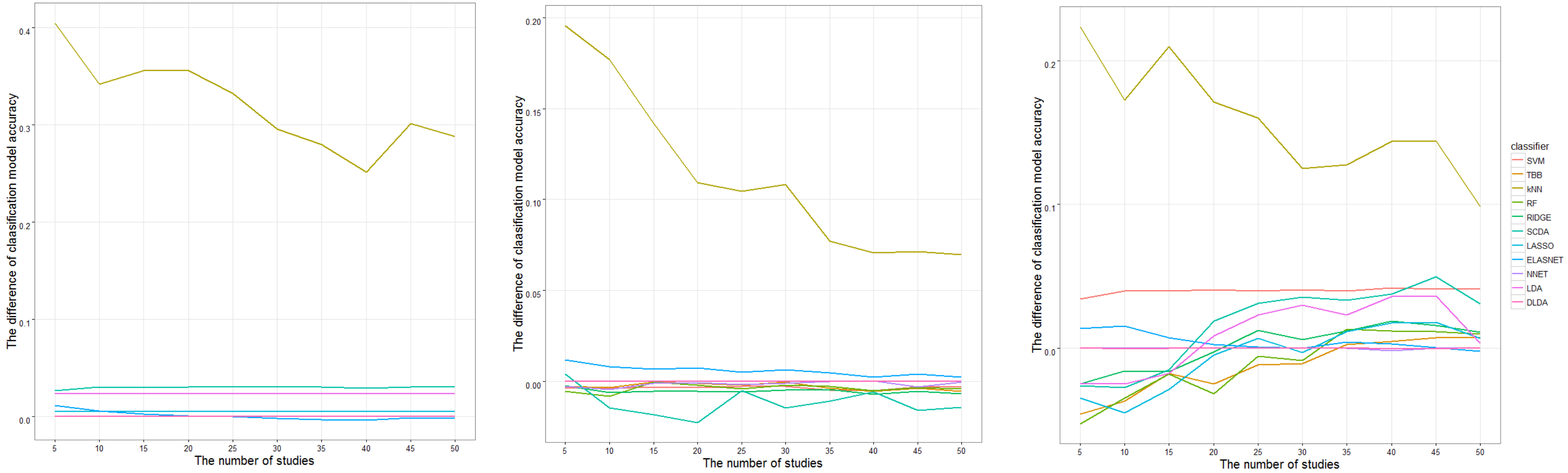

Figure S6. Plot of the difference of classification model accuracies between MA- and individual-classification approach in the simulated datasets, when $\Delta = 0.5, \rho = 0.75$ and (a) $n = 50$ (Simulation 2) (b) $n = 100$ (Simulation 5) (c) $n = 150$ (Simulation 8). The aforementioned simulation parameters resulted in the medium informative datasets.

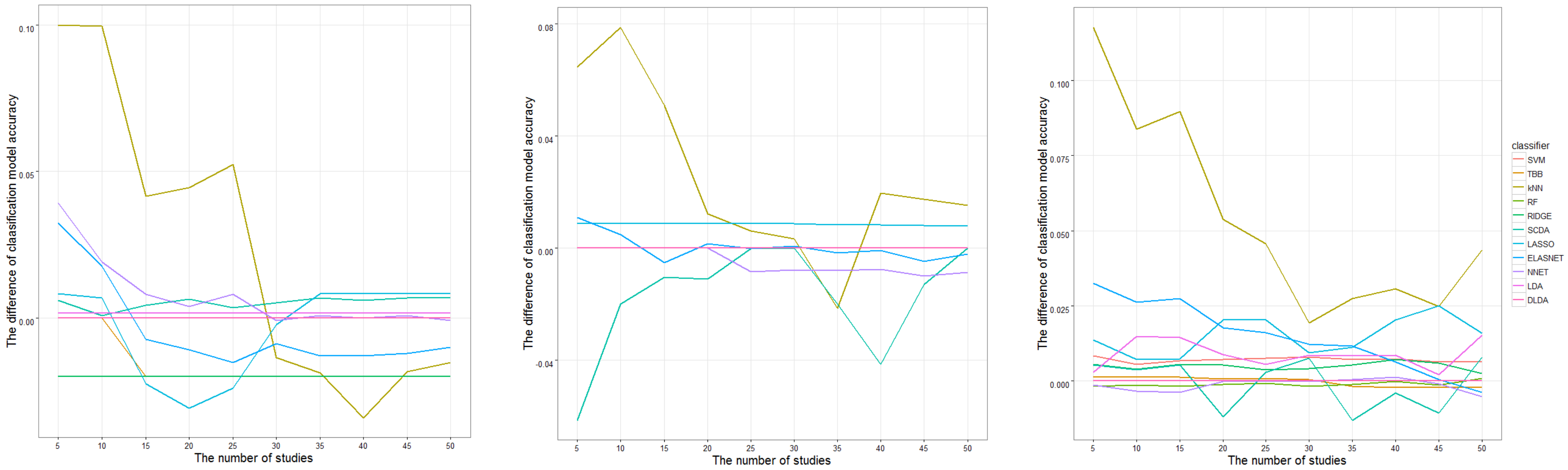

Figure S7. Plot of the difference of classification model accuracies between MA- and individual-classification approach in the simulated datasets, when $\Delta = 0.75, \rho = 0.25$ and (a) $n = 50$ (Simulation 3) (b) $n = 100$ (Simulation 6) (c) $n = 150$ (Simulation 9). The aforementioned simulation parameters resulted in the highly informative datasets.
